# Road Traffic and Childhood Leukemia: The ESCALE Study (SFCE)

**DOI:** 10.1289/ehp.1002429

**Published:** 2010-12-08

**Authors:** Alicia Amigou, Claire Sermage-Faure, Laurent Orsi, Guy Leverger, André Baruchel, Yves Bertrand, Brigitte Nelken, Alain Robert, Gérard Michel, Geneviève Margueritte, Yves Perel, Françoise Mechinaud, Pierre Bordigoni, Denis Hémon, Jacqueline Clavel

**Affiliations:** 1 INSERM (Institut National de la Santé et de la Recherche Médicale), CESP (Centre de recherche en Epidémiologie et Santé des Populations) Environmental Epidemiology of Cancer, Villejuif, France; 2 Université Paris-Sud 11, UMRS-1018, Villejuif, France; 3 AP HP (Assistance Publique des Hôpitaux de Paris), Hôpital Armand Trousseau, Paris, France; 4 AP HP (Assistance Publique des Hôpitaux de Paris), Hôpital Saint-Louis and Hôpital Robert-Debré, Paris, France; 5 Hôpital Debrousse, Lyon, France; 6 Université Lille Nord de France, Lille, France; 7 Hôpital des Enfants, Toulouse, France; 8 Hôpital La Timone, Marseille, France; 9 Hôpital Arnaud de Villeneuve, Montpellier, France; 10 Hôpital Pellegrin Tripode, Bordeaux, France; 11 Hôpital Mere-Enfant, CHU-Nantes, France; 12 Hôpital d’enfants de Brabois, Nancy, France; 13 National Registry of Childhood Blood Malignancies, Villejuif, France

**Keywords:** acute leukemia, benzene, children, environment, epidemiology, road traffic

## Abstract

**Background:**

Traffic is a source of environmental exposures, including benzene, which may be related to childhood leukemia.

**Objectives:**

A national registry–based case–control study [ESCALE (Etude Sur les Cancers et les Leucémies de l’Enfant, Study on Environmental and Genetic Risk Factors of Childhood Cancers and Leukemia)] carried out in France was used to assess the effect of exposure to road traffic exhaust fumes on the risk of childhood leukemia.

**Methods:**

Over the study period, 2003–2004, 763 cases and 1,681 controls < 15 years old were included, and the controls were frequency matched with the cases on age and sex. The ESCALE data were collected by a standardized telephone interview of the mothers. Various indicators of exposure to traffic and pollution were determined using the geocoded addresses at the time of diagnosis for the cases and of interview for the controls. Indicators of the distance from, and density of, main roads and traffic nitrogen dioxide (NO_2_) concentrations derived from traffic emission data were used. Odds ratios (ORs) were estimated using unconditional regression models adjusted for potential confounders.

**Results:**

Acute leukemia (AL) was significantly associated with estimates of traffic NO_2_ concentration at the place of residence > 27.7 μg/m^3^ compared with NO_2_ concentration < 21.9 μg/m^3^ [OR = 1.2; confidence interval (CI), 1.0–1.5] and with the presence of a heavy-traffic road within 500 m compared with the absence of a heavy-traffic road in the same area (OR = 2.0; 95% CI, 1.0–3.6). There was a significant association between AL and a high density of heavy-traffic roads within 500 m compared with the reference category with no heavy-traffic road within 500 m (OR = 2.2; 95% CI, 1.1–4.2), with a significant positive linear trend of the association of AL with the total length of heavy-traffic road within 500m.

**Conclusion:**

This study supports the hypothesis that living close to heavy-traffic roads may increase the risk of childhood leukemia.

Leukemia is the most common childhood cancer, and there are about 470 new cases each year in France (Clavel et al. 2004; Lacour et al. 2010). Except for high doses of ionizing radiation, Down syndrome, a few rare genetic disorders, and certain chemotherapies, which explain few cases, the etiology of childhood leukemia remains largely unknown (Anderson et al. 2006; Buffler et al. 2005; McNally and Parker 2006).

Road traffic is a source of environmental exposure to aromatic compounds, particularly low doses of benzene (Duarte-Davidson et al. 2001; Smith and Zhang 1998). In adults, occupational exposure to high doses of benzene is an established cause of acute leukemia, especially acute nonlymphoblastic leukemia (ANLL). In 1982, the International Agency for Research on Cancer (IARC) assigned benzene to group 1 of the agents carcinogenic to humans. Diesel and gasoline exhaust fumes were classified as probably (group 2A) and possibly (group 2B) carcinogenic to humans (IARC 1989), respectively. A few studies have investigated the role of road traffic in the risk of childhood leukemia, using a variety of traffic indicators to assess exposure.

The indicators were proximity to heavy-traffic roads in two ecological studies and one case–control study, which found no association between acute leukemia (AL) and the presence of a heavy-traffic road within 50 m (Steffen et al. 2004; Visser et al. 2004) or 100 m of the residence (Harrison et al. 1999); daily road traffic in three case–control studies, assessed as the daily number of cars on the road of residence (Savitz and Feingold 1989) or on the road with the highest daily traffic within < 165 m (Reynolds et al. 2001), or weighted by the road–dwelling distance (Langholz et al. 2002; Pearson et al. 2000), which reported a positive association, significant in two studies (Pearson et al. 2000; Savitz and Feingold 1989); traffic density within 150 m in two case–control studies (Reynolds et al. 2004; Von Behren et al. 2008) or traffic density at the residence in another case–control study (Raaschou-Nielsen et al. 2001), with no association; and an ecological study, which found a weak significant association (Reynolds et al. 2002).

A more indirect indicator, the number of cars owned in the area, was associated with leukemia in one ecological study (Nordlinder and Jarvholm 1997) but not in another (Reynolds et al. 2002). Estimates of nitrogen dioxide (NO_2_) concentration were used in three case–control studies (Feychting et al. 1998; Raaschou-Nielsen et al. 2001; Weng et al. 2009), and estimates of benzene concentration were used in two case–control studies (Crosignani et al. 2004; Raaschou-Nielsen et al. 2001) and one ecological study (Whitworth et al. 2008).

All the studies based on pollutant estimates tended to show an association with leukemia, except the Danish study (Raaschou-Nielsen et al. 2001), which was based on particularly reliable data estimated by inclusion of measured NO_2_ and benzene concentrations, traffic patterns, and many other parameters in a model. In addition, a validation study of exposure assessment was conducted on a subgroup. Two case–control studies (Brosselin et al. 2009; Steffen et al. 2004), including our current study, reported positive and significant associations between AL and living next to a gas station. A further case–control study (Harrison et al. 1999) found a positive but nonsignificant association with gas stations within 100 m of the residence.

The national population-based case–control study, ESCALE [Etude Sur les Cancers et les Leucémies de l’Enfant (Study on Environmental and Genetic Risk Factors of Childhood Cancers and Leukemia)] has been used to investigate the relationship between the risk of childhood AL and various indicators of exposure to traffic derived from the address of the subject at the time of the study.

## Materials and Methods

The ESCALE study is a national study conducted in 2003 and 2004 in mainland France (11 million children < 15 years of age) to investigate the role of infectious, environmental, and genetic factors in four childhood neoplastic diseases (acute leukemia, lymphoma, neuroblastoma, and brain tumor) (Brosselin et al. 2009; Rudant et al. 2007, 2008, 2010). In this article we focus on AL.

### Case and control ascertainment

#### Cases

The cases were identified directly by the investigators of the French National Registry of Childhood Blood Malignancies (RNHE) (Clavel et al. 2004) assigned to each pediatric oncology hospital department (Appendix 1). Eligible cases were children first diagnosed with one of the cancers under study between 1 January 2003 and 31 December 2004, age < 15 years, and residence in mainland France at the time of diagnosis. Cases who had been adopted, whose biological mother had died, whose mother did not speak French, or whose mother presented with a serious psychiatric disorder were not eligible. For ethical reasons, the mothers of children who had died or who were receiving hospital palliative care were not contacted. Of the 937 cases of AL identified during the study period, 842 cases were eligible. The reasons for noneligibility consisted of the death of the child (34 cases), hospital palliative care (7 cases), death of the biological mother (10 cases), non-French-speaking mother (29 cases), and mother with a serious psychiatric disorder (15 cases). In all, 763 cases [645 cases of acute lymphoblastic leukemia (ALL), 102 of acute myeloblastic leukemia (AML), and 16 of undifferentiated or biphenotypic AL] of the 842 eligible cases consented to participate (91%). The cases were confirmed, documented, and classified by leukemia cytological and immunological subtype by the RNHE.

#### Controls

Using a quota-sampling method, we randomly selected the controls from the French population over the years 2003 and 2004. We randomly extracted a base of 60,000 phone numbers representative of French telephone subscribers from the national telephone directory. This set was representative of the population in terms of the administrative regions and urbanization. By incrementing each number by 1, we generated a new set of 60,000 numbers, which included unlisted numbers and had geographic and demographic distributions similar to those of the initial set (same first six digits, which determine the location of the line). Quotas were applied to make the age and sex distribution of the controls similar to that of all ESCALE cases, based on estimates from the RNHE (Clavel et al. 2004) and the Regional Childhood Cancer Registries (Desandes et al. 2004), with age group quotas 0–1, 2, 3, 4, 5–6, 7–8, 9–11, and 12–14 years. The number of children < 15 years of age in the household was forced to reflect that of the population by using quotas to prevent a bias in the distribution of birth order, which may happen if the probability of a control being selected among phone subscribers depends on the size of the sibship. We obtained the expected number of children < 15 years of age living in the household for a given age from the 1999 population census [INSEE (National Institute for Statistics and Economic Studies) 1999]. Thus, there were 48 quota strata of age (8 strata), sex (2 strata), and number of children by household (1, 2, 3, or more). The controls were children who were free from cancer, had not been adopted, whose biological mother was alive, free from serious psychiatric disorders, and French-speaking. Of the 50,217 phone numbers dialed, 22,584 did not connect to a household, 24,411 connected to ineligible households, and 862 connected to respondents who hung up before eligibility could be checked. The 2,360 remaining numbers were considered to be those of eligible households, 679 of which refused to participate. Thus, 1,681 mothers were interviewed (71.2%).

### Data collection

Using structured questionnaires, the same trained interviewers carried out the telephone interviews with the biological mothers of the cases and controls. Half of the mothers of the cases were interviewed < 4 months after the diagnosis (range: 1–24 months). The telephone questionnaire elicited information on demographic and socioeconomic characteristics, childhood medical history, childhood environment, lifestyle, and residential history. The interviews also elicited parental occupational history, maternal exposure, and familial history of cancer, allergy, and autoimmune disease. For each residence inhabited since the conception of the index child, the mothers were asked the name of the municipality and its ZIP/area code, type of housing (apartment, house, or farm) and whether a business adjoined it. Only the exact address of the last residence was collected. The degree of urbanization of the municipalities of residence (rural: < 5,000 inhabitants; mixed: 5,000–100,000 inhabitants; urban: > 100,000 inhabitants) was derived from the 1999 census data. Professional category of the parents was the higher of the maternal and paternal occupations at interview and was coded using the two-digit ILO classification (International Labour Organization 1988). This variable was used as an indicator of socioeconomic status (SES).

### Exposure assessment

We used a geographic information system (Mapinfo, Pitney Bowes Software Inc., Troy, NY, USA) to generate the Lambert II coordinates of the residence at the time of diagnosis or interview and automatically match the address with the Navteq (Navteq, Paris, France; http://www.navteq.com/) vector map of the road network. Whenever possible, we manually located addresses that could not be automatically linked. At the end of the process, 2.4% of the subjects could not be located more precisely than by their municipality of residence. Because no quantitative indicators of road traffic on a national scale are available, Navteq function classes were used to characterize the roads on the basis of their importance in the network: Class 1 consisted of high-speed freeways and bypasses, class 2 of main roads connecting class 1 roads, and class 3 of secondary roads. Class 4 and 5 roads, consisting of moderate-speed roads connecting neighborhoods, were not considered in this study. National estimates of NO_2_ concentrations were used as indicators of background air pollution. The National Environmental and Energy Agency (ADEME) (Jeannée et al. 2004) provided a smoothed map of annual traffic NO_2_ concentrations, estimated for the year 2000 by a multiple determinant model using road, transport, and emissions data. The map shows NO_2_ concentrations for mainland France on a 4-km^2^ grid.

We derived various indicators. Proximity to main roads was defined as the presence of heavy-traffic roads within 500 m of the residence. The unexposed category indicated the absence of class 1, 2, and 3 roads. The low-exposure category indicated the presence of at least one class 2 road but no class 1 road or of a class 3 road with no class 1 or class 2 road. The intermediate-exposure category indicated the presence of at least one class 1 road but no class 2 road. The high-exposure category indicated the presence of both class 1 and class 2 roads. The density of heavy-traffic roads was defined as the cumulative lengths of class 1 and 2 roads within 500 m of the residence. Cutoffs were defined *a priori*, based on the joint distribution of the road lengths within 500 m. The unexposed category indicated that the lengths of class 1 and class 2 roads were both equal to zero; the low-density category indicated that the length of class 1 roads was < 750 m (25th percentile); the intermediate-density category indicated that the length of class 1 roads was > 750 m (25th percentile) and the length of class 2 roads was < 750 m (25th percentile); the high-density category indicated that the lengths of class 1 and class 2 roads were both > 750 m (25th percentile). The indicator of traffic-related NO_2_ concentration was extracted from the ADEME map. We assigned to each residence the average of the traffic NO_2_ concentrations at the grid square centers located within 3 km, weighted by the inverse of the distances to the grid square centers. The cutoffs were based on the distribution of the NO_2_ concentration. The low-concentration category consisted in concentrations > 50th percentile (12.2 μg/m^3^), the intermediate-concentration category in concentrations between the 50th and 75th percentiles (between 12.2 μg/m^3^ and 16.2 μg/m^3^), and the high-concentration category in concentrations ≥ 75th percentile.

We then constructed composite exposure indicators by crossing the three variables: proximity to heavy-traffic roads, density of heavy-traffic roads, and traffic NO_2_ concentration. The unexposed category consisted of the intersection of the unexposed categories of the three variables; the high-exposure category in the intersection of the high-exposure categories of the first two variables and the high- or intermediate-exposure category of the last variable. The intermediate-exposure category consisted of the remaining exposure combinations.

Case parents were recruited by the physician attending their child. The parents completed a standardized form indicating that they provided informed consent for participation and that their acceptance or refusal would have no impact on the health care of their child. Control parents provided informed consent by telephone.

### Statistical analysis

We estimated odds ratios (ORs) and their 95% confidence intervals (CIs) using unconditional logistic regression models (all AL) or polychotomous logistic regression (AL types) including the stratification variable used for quota sampling (eight age groups for each sex) and the socioeconomic status variable. We also performed the analyses by AL type and 5-year age group. We tested the stability of the results after additional adjustments and/or stratum analysis for maternal and paternal education, type of housing, degree of urbanization of the municipality of residence, and factors previously related to childhood leukemia in the literature and in the ESCALE study (birth order, early common infections (Rudant et al. 2010), preconception paternal smoking (Rudant et al. 2008), maternal domestic use of pesticides during pregnancy (Rudant et al. 2007), and residence next to a gas station (Brosselin et al. 2009).

We computed tests for trend, when appropriate, from categorical variables. The subjects of each class of the categorical variables were assigned the median value of that class, thus creating a new quantitative and discrete variable from the quantitative and continuous variable of interest. We then tested the linear trend by a likelihood ratio test, comparing the model with the newly generated quantitative and discrete variable with the categorical variable. If linearity was not rejected, the *p*-value of the trend was obtained by testing the slope of the quantitative and continuous variable of interest.

The SAS software package (version 9; SAS Institute Inc., Cary, NC, USA) was used for all the analyses.

The research was conducted in accordance with principles of the Declaration of Helsinki (World Medical Association 2004) and complied with all applicable international regulatory requirements including submission to an ethics committee (DGS No. 2003/0259).

## Results

A total of 763 cases were included, consisting of 645 ALL cases (544 common B-cell ALL, 30 mature B-cell ALL, 67 T-cell ALL, and 4 unspecified ALL) and 118 ANLL cases (102 AML and 16 undifferentiated or biphenotypic leukemia).

### Case and control comparability

ALL distribution showed the expected male predominance (54.3%) and incidence peak at 2–6 years of age (54.8%) [see Supplemental Material, Table 1 (doi:10.1289/ehp.1002429)].

The distribution of controls by age and sex was similar to that of the whole ESCALE case group, but the controls were significantly younger than the AL cases, with a mean age (± SD) of 5.2 ± 3.7 for the cases and 5.5 ± 4.3 years for the controls. However, all the strata contained more than one control per case for adjustment, with the most controls per case in the youngest strata. Fathers of the controls had a slightly higher educational level than fathers of the cases, and parents of the controls had a slightly more qualified professional status than parents of the cases (Table 1). SES was therefore included in logistic regressions relating AL to traffic indicators. There was no significant difference between cases and controls with regard to housing or urbanization at the place of residence at the time of interview. The cases had significantly more often moved at least once (65%) than had the controls (54%). Among the controls, 69% of the only and first-born children had moved at least once, whereas this proportion was 52% for second-born, 41% for third-born, and 40% for at least fourth-born. Overall, the case and control coordinates were obtained with a precision of ≤ 100 m for 97% of the cases and 98% of the controls, but the cases were less often located with a precision of ≤ 15 m (73% vs. 79%).

### Exposure determinants

Among the controls, the most-exposed children in terms of proximity and density of main roads were more often in a higher socioeconomic category than the least-exposed children. However, this relationship was true in the less urban (< 100,000 inhabitants) and most urban (≥ 1,000,000 inhabitants) areas, but not in the intermediate strata of rural/urban status, where the least-exposed children belonged to the highest socioeconomic category. The rural/urban status, closely related to population density, was a strong determinant of traffic NO_2_ concentrations, which ranged from 10.1 to 20.4 μg/m^3^ in the rural areas and Paris area, respectively. The traffic NO_2_ concentration indicator was significantly associated with the indicators of the proximity and density of heavy-traffic roads. However, almost 40% of the controls who lived > 500 m from a main road were estimated to be exposed to traffic NO_2_ at an intermediate (23%) or high (16%) level.

### Exposure to road traffic and risk of childhood acute leukemia

AL was associated with the indicators of proximity and density of heavy-traffic roads (Table 2) and, overall, the associations were similar for ALL and ANLL. The presence of class 1 and 2 roads within 500 m and their presence within 300 m were associated with ORs of 2.0 (95% CI, 1.0–3.6; 22 cases vs. 26 controls) and 2.4 (95% CI, 1.0–5.7; 11 cases vs. 10 controls), respectively. The presence of a class 1 road within 100 m was associated with an OR of 3.7 (95% CI, 1.1–12.2; 7 cases vs. 5 controls).

The density of heavy-traffic roads was significantly related to ALL. The OR associated with the risk of AL increased significantly with increasing density.

AL was also significantly associated with the estimated traffic-related NO_2_ concentrations at the place of residence, with a significant positive linear trend (*p* < 0.05).

The variable combining the heavy-traffic road proximity and density with traffic NO_2_ was significantly associated with AL, with an OR of 2.6 (95% CI, 1.2–5.3) for the most-exposed category versus the unexposed category (Table 3).

### Adjustments for potential confounders

The results were unchanged after adjustment for degree of urbanization, type of housing, and factors related to AL in the ESCALE study: birth order, early common infections in childhood (at least one infection per quarter before 1 year of age), maternal pesticide use during pregnancy, and paternal smoking before conception. Exclusion from the analysis of the 11 cases with Down syndrome and the 35 cases and 42 controls having lived in a residence adjoining a gas station did not modify the results.

Results showed the same trends when the analyses were stratified on the urban/rural status of the residence [see Supplemental Material, Table 2 (doi:10.1289/ehp.1002429)]. However, the associations with the proximity and density of heavy-traffic roads were slightly more pronounced in urban areas.

The associations with the indicators of the proximity and density of heavy-traffic roads were also stronger when the analyses were restricted to the children who had never moved [see Supplemental Material, Table 3 (doi:10.1289/ehp.1002429)]. Similarly, restriction of the analysis to the children who had lived for at least 2 years in the house they inhabited at the time of diagnosis or interview (548 cases and 1,282 controls) strengthened all the relationships. Finally, the results were unchanged after exclusion of the cases and controls for whom the precision of the residence coordinates was > 15 m.

## Discussion

Overall, the results suggest that childhood leukemia is related to traffic. For both ALL and ANLL, the relationship was observed for the indicators of proximity or density of main roads and of NO_2_ concentration. Cases were identified using the data collection system of the RNHE, which makes case selection bias at the identification stage unlikely. The case mother participation rate was very high (91%). The main reason for noninclusion was a child’s poor state of health or death. However, the exposure to a heavy-traffic road is unlikely to have been related to the severity of the disease or short-term survival, particularly because the associations were similar for rural, semi-urban, and urban places of residence, in which health care may differ.

According to the data available from the national registry, cases included in the ESCALE study were slightly younger and more often resided in a rural area than cases diagnosed at the same period and not included in ESCALE. They also tended to reside less close to heavy-traffic roads (2.9% vs. 5.3% within 500 m) and be less exposed to NO_2_ (28% vs. 37% above the 70th percentile) than the cases not included. These comparisons give no indication of any overrepresentation of exposed children among the cases that might have generated the observed association.

Controls were randomly selected from the overall population using the national telephone directory. The quota-sampling process successfully ensured that the responding controls had the same distribution as the case group with regard to sex and age and the same distribution as the overall population with regard to region, birth order, and maternal education: 41% and 37% of the ESCALE controls born in 1995, 1998, or 2003 were first- and second-born children compared with 43% and 34% in the French national perinatal surveys (Blondel et al. 1997, 2001, 2006); 43% had graduate mothers, compared with 39% for the newborns of the perinatal surveys. Unlisted telephone numbers were randomly generated to prevent selection bias by exclusion of controls in higher socioeconomic categories. People with only cell phones could not be contacted and included as controls, but the effect of the resulting selection of parents with a landline on residence characteristics is difficult to predict. For the selection to be able to explain the results, unselected controls with only a cell phone would have to reside close to main roads more often than the selected controls with a landline. According to the National Institute for Prevention and Health Education (INPES), owners of only cell phones would be of more modest social status than the landline owners at the time of the ESCALE study (Beck et al. 2007), whereas in the ESCALE study, the highest socioeconomic categories were more exposed than the more modest categories.

Overall, on the basis of the national perinatal survey, the controls were comparable with the French population in terms of social category and parental education. However, parental socioeconomic status appeared to be slightly higher for controls than cases, which may be because of the absence of the eligible controls who refused to participate. Parental socioeconomic status was positively and significantly associated with the traffic indicators. Therefore, deficient selection of more modest households is unlikely to have underrepresented exposed children among controls and overestimated the relationship. In addition, adjustment and stratification on parental socioeconomic status did not change the results. The cases and controls were also similarly distributed in terms of housing and rural/urban status, which determine traffic exposure. The associations remained consistent when the analyses were stratified or adjusted on these variables.

The exposures were derived from residential address, which is not subject to recall bias. The addresses of all the cases and controls were obtained and the geocoding process mainly generated the residential coordinates with a minimum precision of 100 m. However, the proportion of addresses geocoded with the greatest precision (≤ 15 m) was slightly lower for the cases (73%) than for the controls (79%); the difference was small and the results did not change dramatically under the extreme hypothesis that 6% of the cases were exposed instead of unexposed. Moreover, the ADEME map used for the NO_2_ concentration indicator used 4-km grid squares, which makes it less sensitive to geocoding imprecision. Restriction of the analyses to the subjects with the most precise coordinates did not change the results. We assumed that NO_2_ concentrations at the time of the interviews (2003–2004) were correctly estimated by the measurements made in 2000, or, at least, that the residences were correctly classified using those measurements. A departure from this assumption would be nondifferential and would attenuate the associations if these associations were true. The NO_2_ concentration indicator was positively related with the Navteq traffic indicators, in line with a study conducted in California (Reynolds et al. 2002) in which more precise indicators were available and shown to be very correlated. A Danish study (Raaschou-Nielsen et al. 1997) showed that the front-door concentration did not properly reflect personal exposure to NO_2_ in most of the rural areas, where traffic density was negligible. It is noteworthy that, in this study, the ratio of background NO_2_ concentrations to traffic-related NO_2_ concentrations was stable across the urbanization categories.

In this study, municipalities of residence were available for the whole residential history, but only the last address was complete and could be geocoded, which might be a source of misclassification if the relevant exposure period is prenatal or during early childhood. The fact that the results were strengthened after restricting the analyses to children who had never moved or had lived at least 2 years in their last residence suggests that the most recent period may not be the most relevant.

The potential confounding factors related to socioeconomic factors or the type of residence were systematically taken into account by adjustment and also by stratification on these variables. In the same way, the leukemia-related factors in previous studies were considered by adjustment, exclusion, and also stratum-specific analyses.

The indicators used to assess and quantify the exposure to heavy-traffic roads or air pollution vary markedly from one study to another, which makes comparisons between studies difficult. The indicators used in our study are not exactly the same as those in previously published studies. A Taiwanese study (Weng et al. 2009) based on monitoring stations evidenced an association between AL and the highest background NO_2_ concentrations. The association (OR = 2.3; 95% CI, 1.5–3.5) was stronger than the association observed in this study, which may be related to the fact that the Taiwanese study, on average, covered more urbanized areas. A Swedish study (Feychting et al. 1998) used model-based estimates of NO_2_ concentrations as an indicator of traffic exposure in a nested case–control study over a 25-year period and on the basis of 39 cases showed a nonsignificant association between AL and NO_2_ concentration. A Danish study (Raaschou-Nielsen et al. 2001) used validated modeling of traffic-related NO_2_ and benzene concentrations to assess lifelong exposure to traffic and evidenced no association with AL. An Italian study (Crosignani et al. 2004) evidenced an association between the highest estimates of benzene concentration and the risk of childhood leukemia (OR = 3.9; 95% CI, 1.4–11.3) and showed that OR significantly increased with estimated benzene concentration. A U.S. ecological study (Whitworth et al. 2008) used model-based benzene and 1,3-butadiene concentrations as indicators of traffic. The study showed an increased risk of AL in the census tracts with the highest benzene concentrations (relative risk = 1.4; 95% CI, 1.1–1.8) and similar associations with the highest 1,3-butadiene levels.

The studies that investigated the relationship between AL and the proximity of heavy-traffic roads (Harrison et al. 1999; Steffen et al. 2004; Visser et al. 2004) or the number of vehicles next to the residence daily (Langholz et al. 2002; Pearson et al. 2000; Reynolds et al. 2001; Savitz and Feingold 1989) reported positive associations, which were significant in two of the studies (Savitz and Feingold 1989; Visser et al. 2004). The RRs calculated were similar in magnitude to those detected for the highest traffic exposures in our study.

Three studies used traffic density as an indicator of traffic. An ecological study conducted in California (Reynolds et al. 2002) found a weak significant association between the risk of AL and traffic density (OR = 1.2; 95% CI, 1.0–1.4). A case–control study, also conducted in California (Reynolds et al. 2004), showed no association with high levels of traffic density assessed 150 m from the residence of the child at birth. The Northern California Childhood Leukemia study (Von Behren et al. 2008) showed a slight positive nonsignificant association (OR = 1.2; 95% CI, 0.8–1.8).

Other studies, which used vehicle density (Nordlinder and Jarvholm 1997; Reynolds et al. 2002), gas station density (Weng et al. 2009), presence of a gas station in the vicinity (Harrison et al. 1999), or a gas station adjoining the residence (Brosselin et al. 2009; Steffen et al. 2004) as indicators, have shown a positive association with AL and thus also suggest a link between childhood AL and exposure to pollutants emitted by road traffic.

## Conclusion

This study supports the hypothesis that living close to heavy-traffic roads may increase the risk of childhood leukemia. Our study has several assets that reinforce the reliability of its results. It is a registry-based study, with good participation rates, conducted on a national scale (11 million children < 15 years of age) with heterogeneity in housing, rural/urban status and road density. The controls were successfully representative of the French pediatric population in terms of social categories and rural/urban status. The exposure was assessed on an objective basis, using a geographic information system, with comparable quality for all the included children, at least within a given stratum of rural/urban status. The observation of slight dose–risk relationships also argues in favor of a causal association. The issue warrants further research with enhanced ability to trace lifelong exposure to traffic and benzene.

## Figures and Tables

**Table 1:** Distribution of the cases and controls by parental education and professional category, and characteristics of the place of residence at the time of diagnosis (cases) or interview (controls) [*n* (%)].

Social and residential characteristics	Controls (*n* = 1681)	Cases (*n* = 763)	Percent OR (95% CI)^a^
Maternal educational level			

No secondary school	159 (9)	81 (11)	1.2 (0.9–1.6)
Secondary school	500 (30)	246 (32)	1.2 (0.9–1.4)
High school graduation	320 (19)	138 (18)	1.0 (0.8–1.3)
Higher education	701 (42)	298 (39)	1.0 (Ref)

Paternal educational level			

No secondary school	165 (10)	99 (13)	1.6 (1.2–2.2)
Secondary school	662 (40)	313 (41)	1.2 (1.0–1,5)
High school graduation	236 (14)	114 (15)	1.3 (1.0–1.7)
Higher education	601 (36)	229 (30)	1.0 (Ref)

Parental professional category			

Managers, intellectual/intermediate professions	715 (43)	278 (36)	1.0 (Ref)
Administrative and sales workers	477 (28)	224 (29)	1.2 (1.0–1.5)
Service workers	215 (13)	95 (13)	1.2 (0.9–1.5)
Factory/agricultural workers, unemployed	274 (16)	166 (22)	1.5 (1.2–1.9)

Birth order			

1	708 (42)	376 (49)	1.0 (Ref)
2	608 (36)	246 (32)	0.7 (0.6–0.9)
≥ 3	365 (22)	141 (19)	0.7 (0.5–0.8)

Type of home at interview			

Apartment	484 (29)	249 (33)	1.0 (Ref)
House	1,173 (70)	504 (66)	0.9 (0.7–1.0)
Farm	23 (1)	10 (1)	0.8 (0.4–1.7)

Area of residence at interview			

Rural	601 (36)	249 (33)	1.0 (Ref)
Mixed	391 (23)	182 (24)	1.1 (0.9–1.4)
Urban	689 (41)	330 (43)	1.1 (0.9–1.4)

No. of house moves			

None	766 (46)	264 (35)	1.0 (Ref)
1	613 (36)	292 (38)	1.4 (1.1–1.7)
≥ 2	302 (18)	207 (27)	2.4 (1.9–3.1)

Precision of the coordinates			

≤ 15 m	1,320 (79)	558 (73)	1.0 (Ref)
16–100 m	324 (19)	184 (24)	1.3 (1.1–1.6)
> 100 m	37 (2)	21 (3)	1.3 (0.7–4.3)

Ref, reference.

a ORs and 95% CIs estimated by unconditional logistic regression models including the stratification variable age × sex.

**Table 2:** Relationship between childhood leukemia and indicators of proximity and density of heavy-traffic road traffic NO_2_ concentration at the place of residence.

Indicators of exposure to roads and traffic	Navteq function^a^			Controls (*n* = 1,681)	All AL		ALL		ANLL	
	Class 1	Class 2	Class 3		Cases (*n* = 763)	OR (95% CI)^b^	Cases (*n* = 645)	OR (95% CI)^c^	Cases (*n* = 118)	OR (95% CI)^c^
Proximity to main roads (meters)										

Unexposed	≥ 500 m	≥ 500 m	≥ 500 m	672	282	1.0 (Ref)	236	1.0 (Ref)	46	1.0 (Ref)
Low	≥ 500 m ≥ 500 m	≥ 500 m < 500 m	< 500 m	905	422	1.1 (0.9–1.4)	359	1.1 (0.9–1.4)	63	1.0 (0.7–1.5)
Intermediate	< 500 m	≥ 500 m		78	37	1.2 (0.8–1.8)	32	1.2 (0.8–1.9)	5	1.0 (0.4–2.6)
High	< 500 m	< 500 m		26	22	2.0 (1.0–3.6)	18	1.9 (1.0–3.7)	4	2.2 (0.7–6.8)

Density of heavy-traffic roads within 500 m of the residence										

Unexposed	None	None		1,255	547	1.0 (Ref)	462	1.0 (Ref)	85	1.0 (Ref)
Low	Other exposed combinations			345	163	1.1 (0.9–1.4)	138	1.1 (0.9–1.4)	25	1.1 (0.7–1.7)
Intermediate	≥ 750 m	< 750 m		61	34	1.3 (0.8–2.0)	29	1.3 (0.8–2.1)	5	1.3 (0.5–3.3)
High	≥ 750 m	≥ 750 m		20	19	2.2 (1.1–4.2)	16	2.2 (1.1–4.3)	3	2.1 (0.6–7.3)

Traffic-related NO_2_ concentration										

Low (< 12.2 μg/m^3^)				840	337	1.0 (Ref)	287	1.0 (Ref)	50	1.0 (Ref)
Intermediate (12.2–16.1 μg/m^3^)				421	220	1.3 (1.0–1.6)	188	1.3 (1.0–1.6)	32	1.4 (0.9–2.2)
High (≥ 16.2 μg/m^3^)				420	204	1.2 (1.0–1.5)	169	1.2 (1.0–1.5)	35	1.5 (1.0–2.4)
Missing				0	2		1		1	

*p*-Value for trend test						< 0.05				

Ref, reference.

a Class 1 consisted of high-speed freeways and bypass, class 2 of main roads connecting class 1 roads, and class 3 of secondary roads. Class 4 and 5 roads, corresponding to other roads connecting neighborhoods with moderate speed, were not considered in this study.

b ORs and 95% CIs estimated by unconditional logistic regression models including the stratification variable age × sex and socioeconomic status.

c ORs and 95% CIs estimated by polychotomous logistic regression models including the stratification variable age × sex and socioeconomic status.

**Table 3:** Relationship between childhood leukemia and the composite exposure indicators (heavy-traffic road proximity and density and traffic NO_2_ concentration).

Composite group	Combined indicator of exposure to roads and traffic			Controls (*n* = 1,681)	All AL		ALL		ANLL	
	Proximity	Density	Traffic NO_2_		Cases (*n* = 763)	OR (95% CI)^a^	Cases (*n* = 645)	OR (95% CI)^b^	Cases (*n* = 118)	OR (95% CI)^b^
Unexposed	Unexposed	Unexposed	Low	424	166	1.0 (Ref)	139	1.0 (Ref)	27	1.0 (Ref)
Intermediate	Other less-exposed combinations			1,241	581	1.2 (1.0–1.5)	493	1.2 (1.0–1.6)	88	1.2 (0.7–1.8)
High	High	High	High/intermediate	16	16	2.6 (1.2–5.3)	13	2.4 (1.1–5.3)	3	3.1 (0.8–11.6)
Missing				0	0		0		0	

Ref, reference.

a ORs and 95% CIs estimated by unconditional logistic regression models including the stratification variable age × sex and socioeconomic status.

b ORs and 95% CIs estimated by polychotomous logistic regression models including the stratification variable age × sex and socioeconomic status.

**Appendix I:** SFCE investigators of the ESCALE study.

Principal investigator	Hospital	City (France)
André Baruchel	Hôpital Saint-Louis/Hôpital Robert Debré	Paris
Claire Berger	Centre Hospitalier Universitaire	Saint-Etienne
Christophe Bergeron	Centre Léon Bérard	Lyon
Jean-Louis Bernard	Hôpital La Timone	Marseille
Yves Bertrand	Hôpital Debrousse	Lyon
Pierre Bordigoni	Centre Hospitalier Universitaire	Nancy
Patrick Boutard	Centre Hospitalier Régional Universitaire	Caen
Gérard Couillault	Hôpital d’Enfants	Dijon
Christophe Piguet	Centre Hospitalier Régional Universitaire	Limoges
Anne-Sophie Defachelles	Centre Oscar Lambret	Lille
François Demeocq	Hôpital Hôtel-Dieu	Clermont-Ferrand
Alain Fischer	Hôpital des Enfants Malades	Paris
Virginie Gandemer	Centre Hospitalier Universitaire – Hôpital Sud	Rennes
Charlotte Jubert	Hôpital Pellegrin Tripode	Bordeaux
Dominique Valteau-Couanet	Institut Gustave Roussy	Villejuif
Jean-Pierre Lamagnere	Centre Gatien de Clocheville	Tours
Françoise Lapierre	Centre Hospitalier Universitaire Jean Bernard	Poitiers
Guy Leverger	Hôpital Armand-Trousseau	Paris
Patrick Lutz	Hôpital de Hautepierre	Strasbourg
Geneviève Margueritte	Hôpital Arnaud de Villeneuve	Montpellier
Françoise Mechinaud	Hôpital Mère et Enfants	Nantes
Gérard Michel	Hôpital La Timone	Marseille
Frédéric Millot	Centre Hospitalier Universitaire Jean Bernard	Poitiers
Martine Münzer	American Memorial Hospital	Reims
Brigitte Nelken	Université Lille Nord de France	Lille
Hélène Pacquement	Institut Curie	Paris
Brigitte Pautard	Centre Hospitalier Universitaire	Amiens
Yves Perel	Hôpital Pellegrin Tripode	Bordeaux
Alain Pierre-Kahn	Hôpital Enfants Malades	Paris
Emmanuel Plouvier	Centre Hospitalier Régional	Besançon
Xavier Rialland	Centre Hospitalier Universitaire	Angers
Alain Robert	Hôpital des Enfants	Toulouse
Hervé Rubie	Hôpital des Enfants	Toulouse
Nicolas Sirvent	L’Archet	Nice
Christine Soler	Fondation Lenval	Nice
Jean-Pierre Vannier	Hôpital Charles Nicolle	Rouen

SFCE, Société Française de lutte contre les Cancers et Leucémies de l’Enfant et de l’Adolescent.
